# Mucoepidermoid carcinoma of the head and neck: CRTC1/3 MAML 2 translocation and its prognosticators

**DOI:** 10.1007/s00405-021-07039-2

**Published:** 2021-08-17

**Authors:** Stijn van Weert, Birgit I. Lissenberg-Witte, Elisabeth Bloemena, C. René Leemans

**Affiliations:** 1 Department of Otolaryngology- Head and Neck Surgery, Amsterdam University Medical Centers, Vrije Universiteit Amsterdam, De Boelelaan 1117, 1081 HV Amsterdam, The Netherlands; 2 Department of Epidemiology and Biostatistics, Amsterdam University Medical Centers, Vrije Universiteit Amsterdam, Amsterdam, The Netherlands; 3 Department of Pathology, Amsterdam University Medical Centers, Vrije Universiteit Amsterdam, Amsterdam, The Netherlands; 4 Department of Oral and Maxillofacial Surgery/Pathology, Cancer Center Academic Centre for Dentistry Amsterdam (ACTA) and Amsterdam University Medical Centers, Vrije Universiteit Amsterdam, Amsterdam, The Netherlands

**Keywords:** Salivary glands, Parotid gland, Mucoepidermoid carcinoma, Prognosticators, Translocation

## Abstract

**Purpose:**

Mucoepidermoid carcinoma (MEC) of the head and neck is a prevalent malignant salivary gland tumour with a reported good outcome. The aim of this study was to report the outcome in our centre.

**Methods:**

A retrospective chart analysis with survival analyses was performed combined with fluorescence in situ hybridization (FISH) analysis to assess CRTC1/3 MAML 2 fusion gene presence.

**Results:**

Sixty-four cases of MEC were identified. Median age at presentation was 51.4 years with a predominance for parotid gland involvement. Five, 10- and 20- year disease-free survival was 98%, 90% and 68%, respectively. Overall survival was 94%, 90% and 64%, respectively. Local recurrence was seen up to 14 years after primary diagnosis; distant metastases were diagnosed up to 17 years later. The overall recurrence rate was less than 20 per cent. CRTC1/3 MAML 2 fusion gene presence showed no survival benefit.

**Conclusion:**

MEC of the head and neck has a favorable outcome with the exception of high-grade MEC. PNI and nodal involvement are not rare. CRTC1/3 MAML 2 fusion gene presence showed no survival benefit. The tendency for late onset of loco-regional and distant recurrence should not be underestimated.

## Introduction

Mucoepidermoid carcinoma (MEC) is a glandular epithelial neoplasm characterized by mucous, intermediate and epidermoid cells, with sometimes columnar, clear cell and oncocytic features [1]. It is known as one of the most prevalent malignant salivary gland tumours of the head and neck. Besides adenoid cystic carcinoma (ACC) and acinic cell carcinoma (AciCC) it completes the top three most diagnosed salivary gland malignancies. Three histological grades are recognized (low, intermediate and high grade). Perineural invasion (PNI) is occasionally seen and lymph node metastases are considered rare. Besides the high-grade cases, patient with MEC have a reportedly excellent prognosis with associated long-term disease-free survival. Distant metastases (DM) are seldom encountered and the treatment of choice is surgery, followed by postoperative radiotherapy (PORT) when indicated. High-grade (HG) MEC, however, should be considered a distinct subtype within the group of MEC because of its propensity for nodal disease as well as DM [2].

Both major and minor glands are equally involved with a predominance for the parotid gland, the hard palate and buccal mucosa.

The most important problem in grading of MEC is constituted by the intermediate category and the different systems used which may lead to under- or overgrading depending on the systems used with the direct consequence of under- or overtreating the patient [2–4]. Debate exists on which grading scheme to use (modified Healey, AFIP and Brandwein) and which treatment strategy to use [5, 6]. Much like grading in other types of malignant salivary gland cancers, the application of these systems is time-consuming due to the point-based character and prone to inter- observer bias [2].

Nonetheless, grading and clinical stage are historically considered the main predictors of survival in MEC [2, 5–8]. Part of the MEC are characterized by a specific translocation of t(11;19)(q21;p13) leading to CRTC1/3 MAML 2 fusion gene. The presence of this fusion gene was originally reported mostly in low-grade tumours but more recently, it became apparent that also a considerable percentage of intermediate and high-grade tumours bear the translocation [9–11]. The presence of this translocation was initially considered to have a beneficial impact on outcome [12, 13]. More recent research, however, suggests that there is no correlation between tumour status or survival and translocation status [14].

In this analysis, 64 cases of MEC diagnosed and treated at our institution over a 30-year period are reviewed with regard to prognosticators and outcome, including histological grading and translocation analysis.

## Materials and methods

Medical charts of patients diagnosed with MEC from 1984 to 2013 were reviewed. Sixty-four cases of MEC were identified for further analysis. All patients were entirely treated and followed up at our institution. Clinical work up was standardised and consisted of physical examination, ultrasound guided fine-needle aspiration cytology (US-FNAC) or biopsy depending on localisation of the primary lesion and computer tomography (CT) or magnetic resonance (MR) imaging. A chest X- ray was routinely performed at first visit. Follow-up after treatment was done 2 monthly to 6 monthly for the first 5 ears followed by annual control visits over a total of 10- to 20-year period. Staging was done according to the TNM classification of the Union for International Cancer Control (UICC), eighth edition [15].

In all cases surgery with curative intent was feasible, followed by PORT in case of advanced stage disease or adverse features, e.g. perineural invasion (PNI), angio- invasion or unsatisfactory surgical margins.

Variables analysed were age, gender, T- stage, N- status, extracapsular spread (ECS), surgical margins, PNI, tumour grade and PORT.

**Fig. 1:**
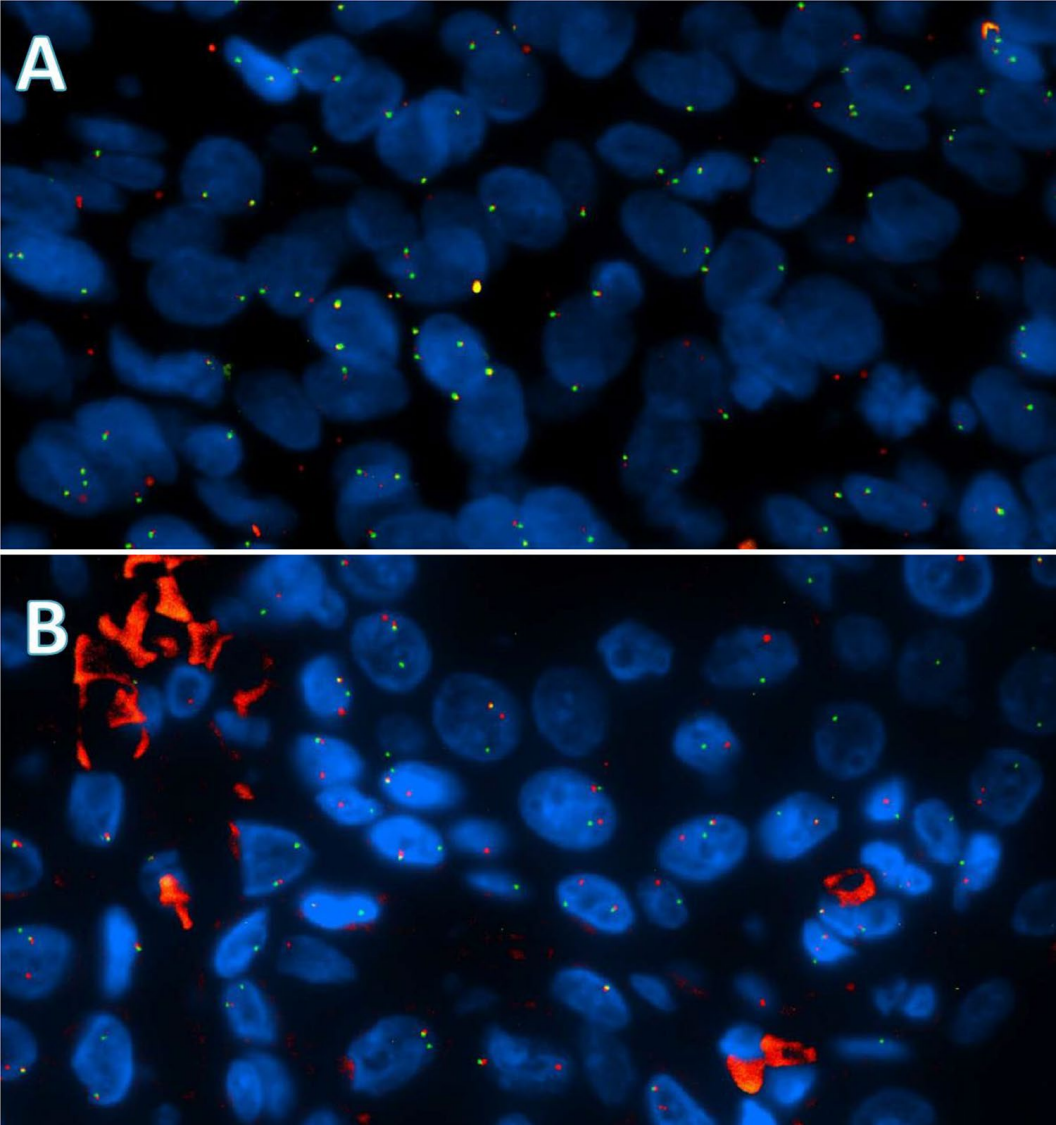
**A** Example of FISH for tumour with a MAML2 translocation. The nuclei of most tumourcells (blue) contain a split signal (red and green dot). **B** Example of FISH for tumour without the translocation. All nuclei show an orange (green–red) unsplit signal

For detection of the translocation in MEC samples, fluorescence in situ hybridization (FISH) analysis was carried out on 4 μm tissue sections according to the manufacturer’s protocol, using ZytoLight^®^ SPEC MAML2 Dual Color Break Apart Probe (ZytoVision Ltd, Bremerhaven, Germany) as described previously [11]. Due to lack of material or poor quality 45/64 tumours could be analysed for translocation status. The MAML2 Dual Color Break Apart Probe can detect rearrangements involving the MAML2 gene irrespective of the fusion partner (including the CRTC3-MAML2 fusion). The nuclei were counterstained with 4′,6-diamidino-2-phenylindole (DAPI), diluted in Vectashield, and samples were evaluated by fluorescence microscopy (ZyGreen: excitation 503 nm, emission 528 nm; ZyOrange: excitation 547 nm, emission 572 nm). Cells without the t(11;19)(q21;p13) translocation show fused green and red signals, typically resulting in a yellow signal. Translocation-positive cells exhibit fused green and red, as well as separated red and green signals, or split signal (Fig. 1A, B). A MEC sample was considered positive for the t(11;19)(q21;p13) translocation when the split signal was identified in at least 10 out of 100 cells. In all but one of 64 cases multiple slides were available for revision by an expert head and neck pathologist (EB). Grade was revised by the point-based AFIP system as suggested by the World Health Organization (WHO) [1]. Survival analyses were done using SPSS statistical software version 22.0 (IBM, New York). Disease-specific survival (DSS) and overall survival (OS) were estimated through Kaplan–Meier curves. Multivariate analysis could not be reliably performed due to the small number of events in this typically indolent type of disease.

## Results

The clinicopathological data are depicted in Table 1. CRTC 1/3- MAML 2 fusion gene analysis was feasible in 42 cases. In 29/42 (69%) cases the translocation was present. Survival analyses showed no significant association nor was there a trend observed with the presence of the translocation. Fusion gene presence relative to tumour grades was 64%, 88% and 56%, respectively, for low, intermediate and high grade. Thirty-three cases of MEC staged N0 were available for translocation analysis showing presence in 24/33 (73%). For N + this was 4/9 (44%).

**Table 1:** Clinicopathological data of 64 cases of MEC

		*N*	%
Age			
Median	51.4		
Range	8–87		
Gender			
Male		35	54
Female		29	46
Site			
Major			
Parotid		26	40
Submandibular		1	2
Sublingual		–	–
Minor			
Hard palate^a^		16	25
Oral cavity (non palate)		6	9
Oropharynx		9	14
Larynx		4	6
Other		2	3
T- stage			
T0		1	1
T1–2		52	81
T3–4		11	17
N-status			
N0		52	82
N +		12	18
ECS		4	33
M- status			
M0		60	94
M1		4	6
UICC (8th edition) stage^16^			
I		33	52
II		12	18
III		7	11
IV		12	18
Surgical margins			
Clear		30	46
Close		24	37
Positive		7	11
Uncertain		3	5
Grade (AFIP)			
Low		38	60
Intermediate		10	15
High		16	25
PNI			
Yes		10	16
No		54	84
CRTC1/3- MAML 2 fusion gene			
Positive		28	44
Negative		13	20
N/A		23	36
PORT			
Yes		43	66
No		21	33

*UICC* Union for International Cancer Control, *PNI* Perineural invasion, *PORT* postoperative radiation therapy, *N/A* not assessed

^a^Two cases with both hard and soft palate involvement

Median age at presentation was 51.4 years (range 8- 87). Taking this median as a cut-off, it showed that patients aged 51 or more suffer a significantly worse DSS. It should be noted that advanced stage disease was more prominent in this group (≤ 50: 7% vs > 50: 42%). There is a trend for worse disease-free survival in older patients when analysed for early stage (T1–2) disease alone (*p* = 0.051). There was no clear gender predilection (54% male) and no difference in survival between males and females. Median follow-up was 102 months with a maximum of 258 months. The majority of MEC was located in the minor salivary glands (58%).

All but one (submandibular gland) major gland tumours were located in the parotid gland; the hard palate (palate: *n* = 19: 15 hard, 2 soft, 2 combined) was the most affected site amongst the minor gland tumours (50%). 40% of tumours involved the parotid gland. Survival analysis showed no difference for minor versus major gland involvement.

The vast majority (82%) of tumours were early stage (T1–2) tumours at the time of diagnosis.

**Fig. 2:**
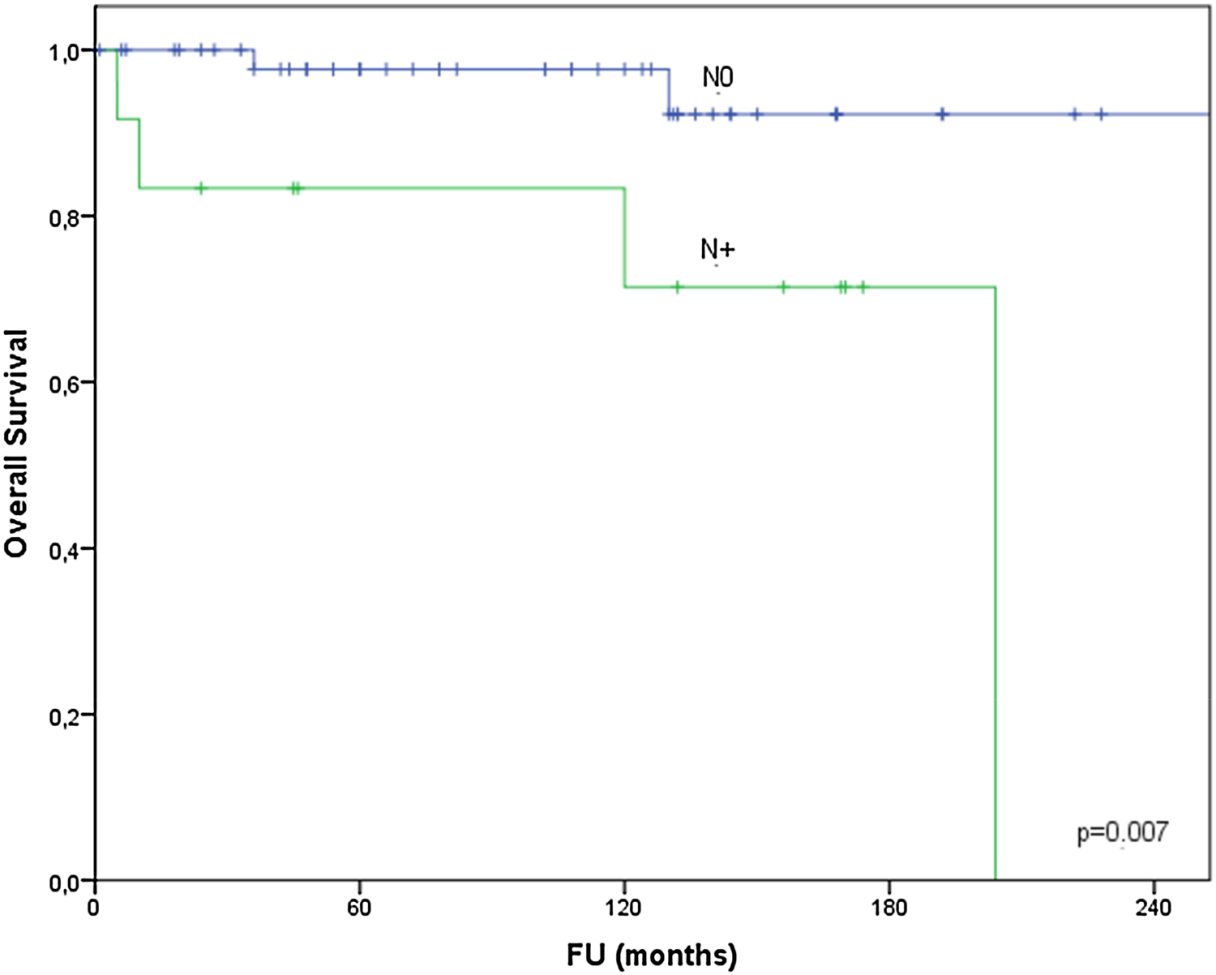
Overall survival relative to N- status

Nodal involvement was seen in 18% of cases of which four showed extracapsular spread (ECS) after surgical excision. N + disease was diagnosed in 11% of early stage tumours and in 42% of T3–4 tumours. The distribution of N- status was N1 (*n* = 7; 5 parotid, 1 oropharynx and 1 palate), N2b (*n* = 3; parotid, submandibular and oropharynx), N2c (*n* = 1; oropharynx midline) and N3 (*n* = 1; unknown primary). Relative to grade nodal disease was seen in 11%, 30% and 31% for low, intermediate and high-grade (HG) MEC, respectively. N + disease was negatively associated with DSS, OS (Fig. 2) and with developing distant disease as shown in Table 2.

**Table 2:** 5-, 10- and 20-year survival rates (%) in the current study (*n* = 64)

	LCR	DSS	DFS	DDFS	OS
5y (%)	95	94	98	98	94
10y (%)	86	94	90	92	90
20y (%)	65	67	68	69	64
Age > 50y (*p* value)	.**023**	**.030**	.061	.06	**.017**
UICC Stage (Early vs advanced) (*p* value)	.19	.075	.11	.28	**.026**
Grade (*p* value)					
Low vs intermediate (*p* value)	.53	.69	.86	.71	.69
Intermediate vs high (*p* value)	.58	.51	.32	.86	.25
Low-intermediate vs high (*p* value)	.17	.069	.11	.31	**.011**
N + (*p* value)	.63	**.032**	.17	**.010**	**.007**
PNI (*p* value)	.064	**.026**	**.027**	.16	**.047**
Surgical margins (*p* value)	.998	.33	.36	.095	.56
CRTC 1/3- MAML 2 fusion gene (*p* value)	.55	.10	.094	.098	.29
PORT (*p* value)	.83	.14	.99	.20	.11
Major/minor (*p* value)	.42	.90	.54	.58	.85

All values in bold are significant *p*- values with a cut off of *p*<.05

*LCR* local control rate, *DSS* disease-specific survival, *DFS* disease free survival, *DDFS* distant disease-free survival, *OS* overall survival, *UICC* Union for International Cancer Control, *N* + nodal involvement status, *PNI* perineural invasion, *PORT* postoperative radiotherapy

Distant disease was diagnosed in four (6%) cases, two of which were in the preoperative work up (both high grade). Two patients developed distant disease after a prior local recurrence (one low grade (translocation status negative); one intermediate (translocation status positive). In one case the interval between the local recurrence and diagnosis of DM was 6 years.

Surgical margins were defined as clear (≥ 5 mm), close (1 > 5 mm) and positive (≤ 1 mm). Margins were clear in 46%, close in 31% and positive in 14% of cases with no association with T- status. Margins for parotid MEC were clear in 35% and close in 50% of cases and clear in soft/hard palate in 63% with the remainder of cases showing only close margins..

A close or positive margin status was not negatively associated with outcome.

**Fig. 3:**
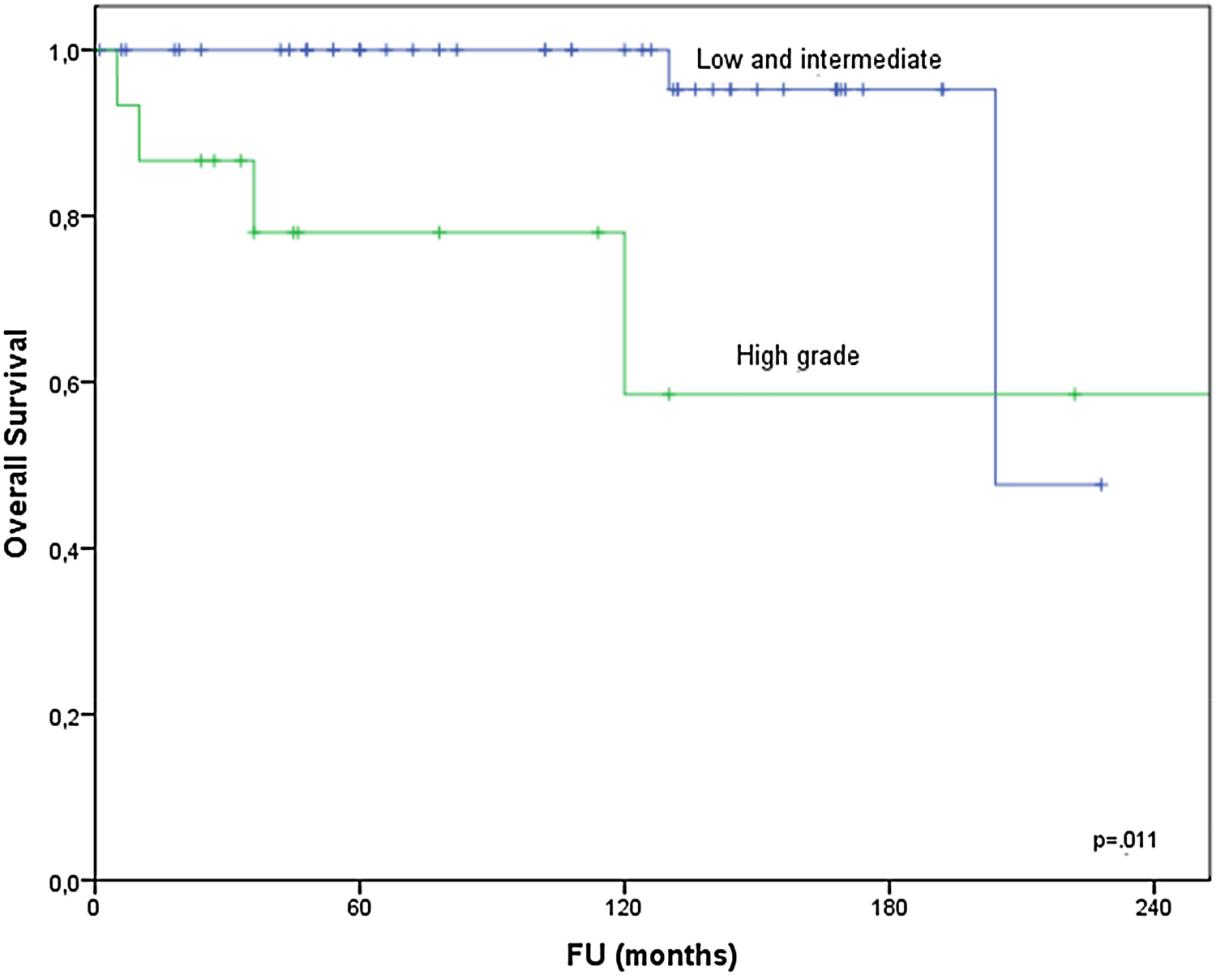
Overall survival relative to grade

With regard to histological grade the incidence of low-grade MEC was highest with 60%. The incidence of intermediate and HG MEC was 15% and 25%, respectively. HG MEC vs. low/ intermediate grade was negatively associated with OS: 5- and 10- year OS of 100% for low and intermediate grade versus 78% and 59% for high grade, respectively. *p* = 0.011; Fig. 3).

**Fig. 4:**
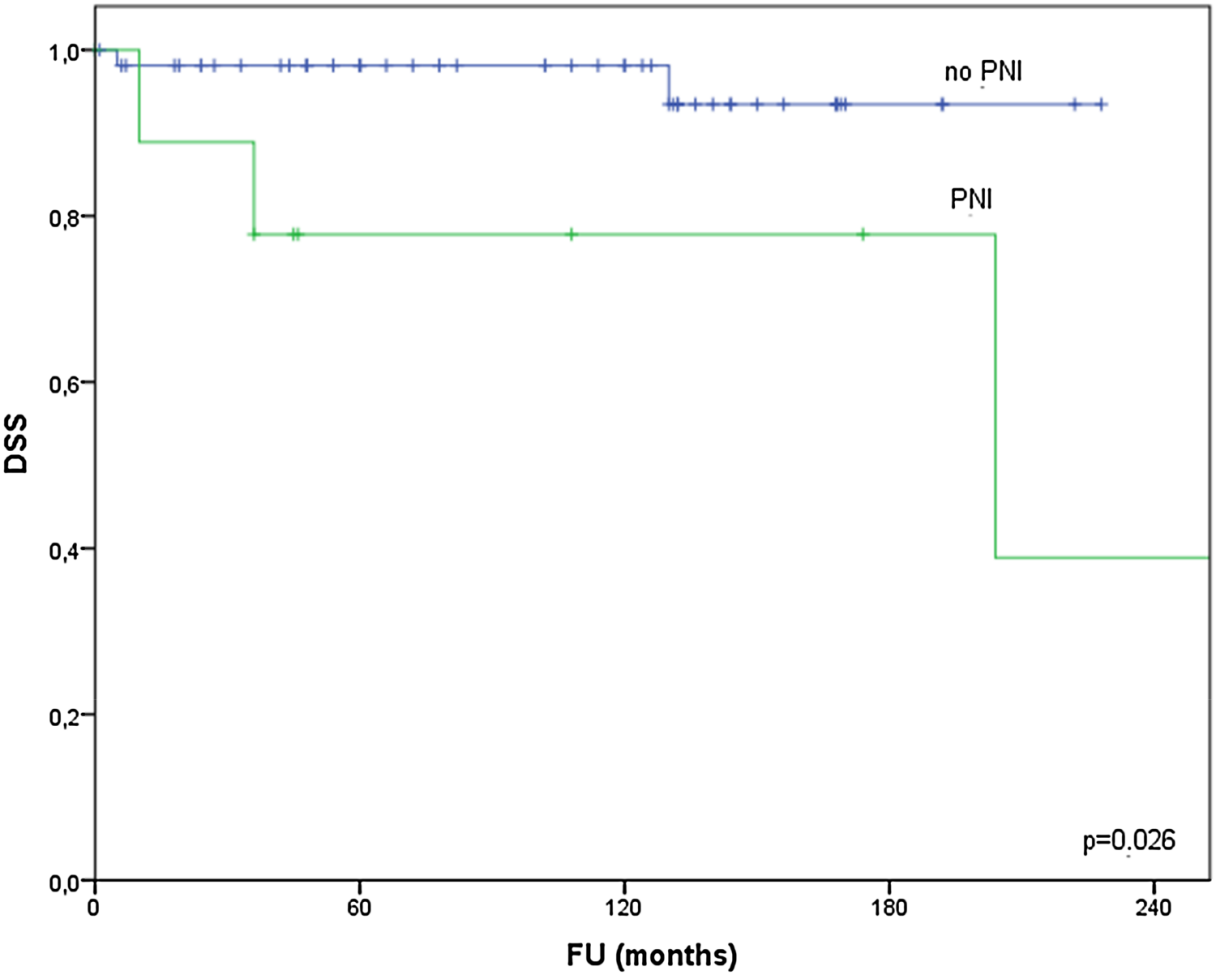
Disease-specific survival relative to PNI

PNI (two points in the AFIP grading system) was diagnosed in 16% of cases and never in case of low-grade MEC. PNI was present in 44% of high-grade tumours and in 30% of intermediate-grade tumours. PNI was negatively associated with DSS (Fig. 4), DFS and OS.

Sixty- three per cent of patients received PORT. Advanced stage tumours (T3–4), high-grade tumours and tumours with PNI all received PORT. In early-stage tumours (T1–2), low-grade tumours and tumours without PNI this was 58%, 47% and 61%, respectively. There was no superior local control or survival benefit in case of PORT. Local control rate was even worse in the group receiving PORT although no significant difference was found.

For the total cohort analyzed, 5, 10- and 20- year DFS was 98%, 90% and 68%, respectively. For OS this was 94%, 90% and 64%, respectively.

**Fig. 5:**
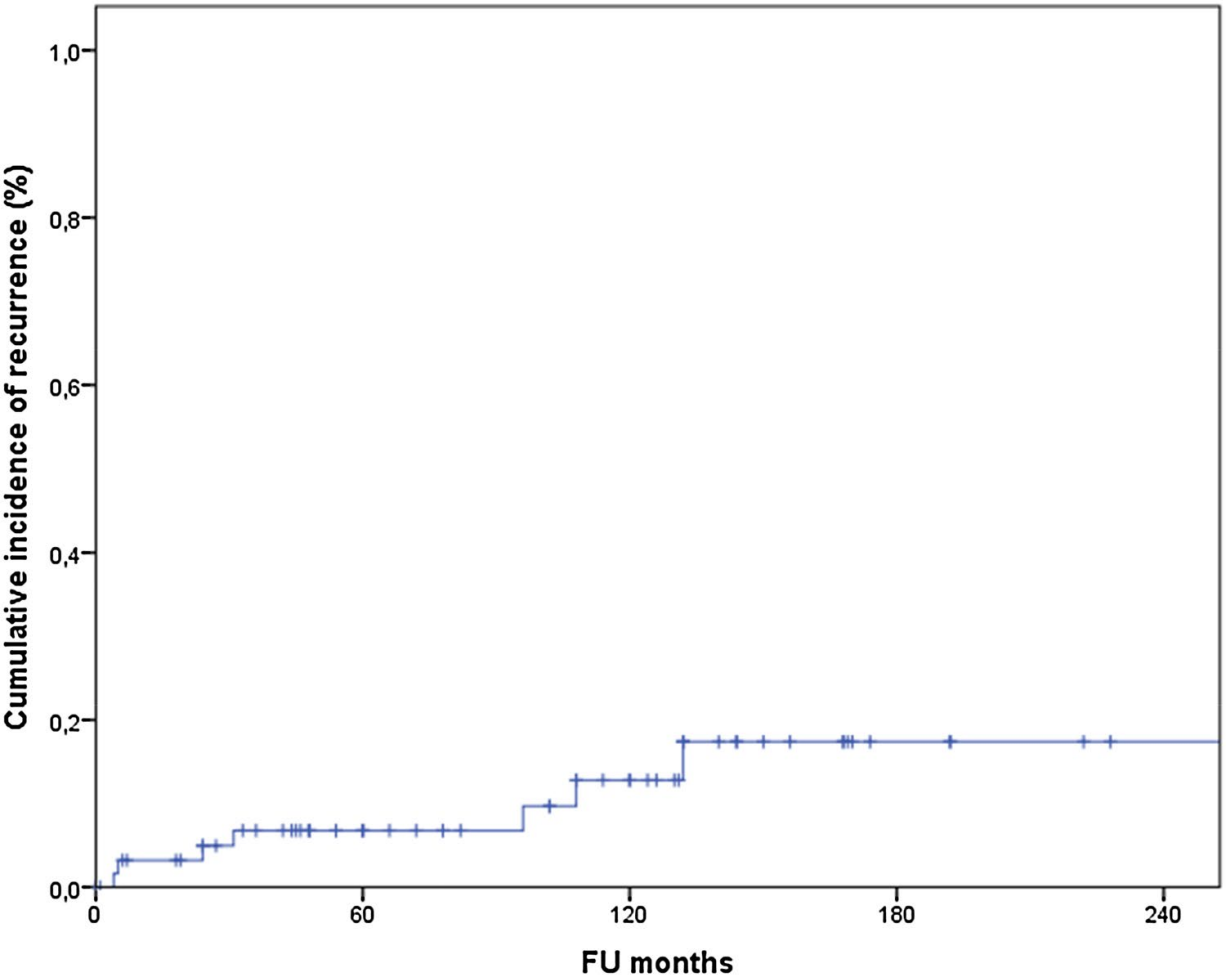
Cumulative incidence of recurrence. In one case a local recurrence was diagnosed prior to distant disease (DM after 204 months). Therefore, this late onset event is not shown in this graph. Less than 20% of patients develop recurrent disease

Local recurrence was seen up to 14 years after primary diagnosis where DM were diagnosed up to 17 years later. The overall recurrence rate in MEC was less than 20 per cent. (Fig. 5).

## Discussion

MEC of the head and neck is generally known as a disease with a favourable outcome. Ellis et al. describe a 3:2 female predilection which we could not confirm in the present study [16].

The most reported sites affected by MEC are the parotid gland and the hard palate, which is in accordance with our series [17]. Similar to our findings, early-stage tumours as well as low-grade tumours are predominantly diagnosed [18].

Accuracy of fine-needle aspiration (FNA) is acceptable for HG MEC (MEC is identified as MEC); 87%) but less so for low-grade tumours 68%) where others suggest to perform core needle biopsy (CNB) which has a higher accuracy than FNA for detecting salivary gland malignancy in general [19, 20]. The risk of seeding is almost negligible for both procedures [21]. Ample cytological experience with MEC ultimately leads to superior interpretation of FNA [22].

According to Kashiwagi et al. pre- operative MR imaging in case of suspicion of MEC may show different characteristics depending on grade [23]. With the current use of diffusion weighted imaging (DW MRI) the distinction between benign and malignant salivary gland lesions has dramatically improved [24]. In case of uncertain cytology or histology this may aid in preoperative planning. CT imaging is not suited for identifying PNI but may aid in evaluating bony erosion or invasion [25]. There is little experience with 18-FDG PET- CT in MEC and previous reports mainly produce data on salivary carcinoma in general with small sample sizes for MEC [26]. In pulmonary MEC a correlation between high standardized uptake value (SUVmax) and HG MEC is suggested [27].

There is no debate regarding the treatment of MEC: surgery with or without PORT. One can discuss the necessity of (elective) neck dissection (END) or its extent as it has been described in a review by Moss et al. [28]. They report on a relatively high incidence of occult nodal disease mainly in relation to high-grade tumours which should justify END in these patients fit for surgery. The problem, however, is that discriminating in grade on pre-operative histopathological/cytological analysis is cumbersome due to the earlier mentioned problems with the grading systems applied [2]. Chen et al. found an incidence (Surveillance, Epidemiology and End Results; SEER) of 34%, 8.1% and 3.3% for high, intermediate and low-grade, respectively, of positive nodes in levels I–III. Based on this they suggest to perform END only in case of HG MEC [29]. In the current study the difference in incidence of nodal disease in intermediate and HG MEC was similar (30% and 31%, respectively). With an overall incidence of positive nodes of 18% in the current series and the possible risk of undergrading it might be a potential risk to refrain from END in all cases which are not HG MEC cases. Apart from this, the different grading systems used for MEC (AFIP, Brandwein and modified Healey) makes grading prone to down- and upgrading as is also discussed by Chen et al. and Seethala et al. [2, 29]. It is unclear which grading schemes were used in all individual cases from the SEER database but the use of multiple schemes might have influenced the incidence of nodal involvement relative to grade. A recent report by Qannam et al. describes the AFIP system (used in this study) as the most suitable [30]. The existence of these three grading systems will continue to contribute to inter-observer variability in the future. Ganly et al. have suggested to merely look for high mitotic rate and necrosis as these two features should predict a poorer outcome [30–32].

Positive or close margin status surprisingly did not show poorer outcomes than cases with clear margins. Achieving clear margins is difficult, mainly in the parotid (35%), oropharynx and oral cavity. We reached a 46% clear margin rate which seems reasonable in comparison to previous reports. McHugh et al., for example describe a 30% clear margin status in their case series of 125 patients [17]. An explanation for the relatively high percentage of close margins (50%) in the current study for MEC of the parotid gland is the proximity of the facial nerve and other surrounding anatomical structures in this area.

Radiotherapy is historically employed as an adjuvant treatment in case of aggressive features (HG MEC), advanced stage disease, PNI, angio- invasion, extra- glandular growth and incomplete surgical margins. The National Comprehensive Cancer Network (NCCN) recommends PORT in early-stage (T1–2) disease in case of spillage, PNI and intermediate/ HG MEC [17, 31, 33].

In this cohort, 66% of patients received PORT, mainly in case of positive or close margins, PNI, high-grade disease, nodal involvement and advanced stage. This probably explains why the PORT- group has no better outcome in survival analyses compared to the no PORT- group; initial prognosis was worse due to adverse features necessitating PORT. Okomura et al. recently reported on possibly refraining from PORT in case of early-stage disease (T1–2) in the presence of the translocation CRTC1/3- MAML2 fusion gene, even in case of intermediate or HG MEC. They reported 4/47 local recurrences which could be locally treated [31]. The results should be interpreted with caution due to the relatively small number of cases.

The CRTC1/3- MAML 2 fusion gene translocation -CRTC1 first described as a candidate gene for induction of salivary gland tumours by Tonon et al. and CRTC3 by Fehr et al. [34, 35]- was also analyzed in our group and was present in 69% of cases. This percentage is in accordance with the data published by Saade et al. who found 56% in their series and reviewed six more studies with an average of 62% [36]. Chenevert et al. found a 100% prevalence of the translocation in their series with a relative small sample size (*n* = 14) [37]. Nevertheless, large differences are seen with regard to HG MEC ranging from 0 to 71%. Again, this is highly probably due to different grading systems used. Saade et al. further confirmed the unique correlation for the CRTC1/3- MAML 2 transcript with MEC which makes it a useful diagnostic feature. No survival benefit was found by Saade et al. in case of presence of the translocation [36]. This is in accordance with our findings. A recent study by Birkeland et al.—analyzing 90 cases of MEC for CRTC 1/3- MAML2 fusion gene—found similar results [14]. This contradicts the potential survival benefit described in previous series. It should be noted that these series were mostly relatively small warranting prospective multi centre studies for proper analysis [12, 36–38].

MEC of the head and neck has a favourable prognosis in general. HG MEC, however, should be considered a specific subtype with higher incidence of nodal and distant disease leading to poor overall survival. PNI and nodal involvement seem to be strong negative prognosticators and are relatively frequently encountered (15% and 18%, respectively).

The mainstay of treatment is still surgery with PORT when indicated. To date, there are no effective adjuvant systemic treatments for MEC. There have been reports on partial responses from cisplatin, paclitaxel and gemcitabine, but these treatments have not been considered standard of treatment in the recurrent/ metastatic setting [39]. The CRTC1/3- MAML 2 fusion gene translocation might be a target for adjuvant systemic treatment in the future. Other genetic alterations, such as deletion in the CDKN2A/ p16 gene, might be worth exploring in this respect [39–42]. Treatment for patients in the recurrent/ metastatic phase should be optimized in the future.

Treating physicians should be aware of the potential of MEC for late onset local and distant recurrence and the distinct and possible relentless course of HG MEC.

## Data Availability

Available upon request

## Code availability

SPSS statistical software.
